# There is no happiness in positive affect: the pervasive misunderstanding of the rotated circumplex model

**DOI:** 10.3389/fpsyg.2024.1301428

**Published:** 2024-03-28

**Authors:** Lisa R. LaRowe, Lauren Connell Bohlen, David M. Williams

**Affiliations:** ^1^Massachusetts General Hospital, Harvard Medical School, Boston, MA, United States; ^2^School of Public Health, Brown University, Providence, RI, United States

**Keywords:** positive affect, negative affect, circumplex model, valence, arousal

## Abstract

Research on *positive affect* (PA) and *negative affect* (NA) is often guided by the rotational variant of the circumplex model of affect (RCMA). According to the RCMA, PA and NA are posited to be orthogonal, with PA ranging from the union of positive valence and high activation (e.g., excited) to the union of negative valence and low activation (e.g., sluggish), and NA ranging from the union of negative valence and high activation (e.g., distressed) to the union of positive valence and low activation (e.g., relaxed). However, many authors incorrectly interpret the RCMA as positing that positively valenced affect (i.e., pleasure) and negatively valenced affect (i.e., displeasure)—rather than PA and NA, as defined in the RCMA—are orthogonal. This “received view” of the RCMA has led to significant confusion in the literature. The present paper articulates the “received view” of the RCMA and characterizes its prevalence in psychological research. A random sample of 140 empirical research articles on affect published in 14 high-impact journals covering a range of psychological subdisciplines were reviewed. Over half of the articles subscribing to the RCMA showed evidence of the “received view,” demonstrating that misuse of the terms PA and NA in the context of the RCMA is rampant in the psychological literature. To reduce continued confusion in the literature, we recommend abandoning use of the terms *positive affect* and *negative affect*. We further recommend referring to the two dimensions of the RCMA as *positive activation* and *negative activation*, and the two poles of the valence dimension as *positive valence* and *negative valence* (or pleasure and displeasure).

## Introduction

Research on *positive affect* (PA) and *negative affect* (NA) is pervasive and growing in psychological science. Reference to these terms in abstracts of peer-reviewed journal articles has increased steadily, from 246 in 2000 to 1,251 in 2021 according to the PsycInfo Database. There is also, however, pervasive ambiguity in the conceptualization and interpretation of findings on PA and NA that threatens to hinder growth in the field of affective science and across multiple psychological subdisciplines.

Researchers often conceptualize PA and NA in the context of the foundational paper by Watson and Tellegen (1985) that described a rotational variant of the affect circumplex model, referred to hereafter as the rotated circumplex model of affect (RCMA). According to the RCMA, the PA dimension ranges from the union of positive valence and high activation (e.g., excited) to the union of negative valence and low activation (e.g., sluggish), and the NA dimension ranges from the union of negative valence and high activation (e.g., distressed) to the union of positive valence and low activation (e.g., relaxed). The RCMA also proposes that PA and NA are theoretically orthogonal and, thus, are hypothesized to be empirically uncorrelated. Consistent with the RCMA, empirical research on PA and NA often employs the Positive Affect and Negative Affect Schedule (PANAS) (Watson et al., 1988) for assessment of PA and NA.

However, definitions of PA and NA and interpretations of empirical findings from the PANAS are often inconsistent with the major tenets of the RCMA. Specifically, contrary to the RCMA, researchers often define and/or interpret the PA and NA dimensions and corresponding PA and NA scales of the PANAS as though they encompass all positively valenced and negatively valenced affective states, respectively. When combined with the RCMA principle that PA and NA are orthogonal, the resulting interpretation is that *positively valenced affect (i.e., pleasure) and negatively valenced affect (i.e., displeasure) are theoretically orthogonal and therefore should be empirically uncorrelated*. Indeed, the latter has become the “received view” of the RCMA, despite the fact that it is inconsistent with the RCMA and the development and proper interpretation of the PANAS (Table 1).

**Table 1:** The “received view” of the rotated circumplex model of affect (RCMA).

	Correct interpretation of the RCMA	“Received view” of the RCMA
Conceptualization of affect	*Positive affect* ranges from high activation, positive valence (e.g., excited) to low activation, negative valence (e.g., sluggish) *Negative affect* ranges from high activation, negative valence (e.g., distressed) to low activation, positive valence (e.g., relaxed)	*Positive affect* includes all positively valenced affective states (e.g., excited, relaxed, happy) *Negative affect* includes all negatively valenced states (e.g., distressed, sluggish, sad)
Principal of orthogonality in the RCMA	*Positive affect* (e.g., excited) and *negative affect* (e.g., distressed), as they are defined in the RCMA, are theoretically orthogonal and therefore should be empirically uncorrelated.	*Positively valenced affective states* (e.g., happy) and *negatively valenced affective states* (i.e., sad) are theoretically orthogonal and therefore should be empirically uncorrelated.

The misinterpretation of the RCMA has been documented previously, first in a series of papers by Watson (author of the seminal paper on the RCMA; Tellegen et al., 1999; Watson and Tellegen, 1999; Watson et al., 1999) and Russell [author of the foundational paper on the unrotated circumplex model of affect (UCMA); Russell and Barrett, 1999; Russell and Carroll, 1999a,b; Yik et al., 1999], and then in papers by Ekkekakis and Petruzzello (2002), Ekkekakis (2008), and Ekkekakis and Zenko (2016). The purpose of the present paper is to describe the problem in further detail, and to empirically evaluate the pervasiveness of the *“*received view*”* in the psychological literature.

First, we review the major tenets of the RCMA, including its similarities and differences with the UCMA, and we discuss the common errors in understanding the RCMA. We liberally quote from the authors of the RCMA (with italics from the original retained in all cases) to allow readers to directly evaluate and interpret their meaning. Second, we use a systematic approach to quantifying frequency of the “received view” in reports of empirical research on PA and NA. Though not a systematic review in the traditional sense, because we are not summarizing *empirical findings* across studies, we nonetheless take a systematic approach to characterizing the prevalence of the misunderstanding of the RCMA (i.e., the *“*received view*”*). Finally, we discuss the theoretical and practical implications of the findings from the review and provide recommendations to avoid misunderstandings in future research.

## The circumplex models of affect

Affect is used herein as an umbrella term encompassing all valenced states, including core affect (e.g., pleasure, displeasure), moods (e.g., depressed, irritable, cheerful), and emotions (e.g., joyful, angry, embarrassed) (e.g., Russell, 2003). The circumplex models of affect posit that discrete moods and emotions are a function of anywhere from one to 11 dimensions of affect (for reviews, see Russell, 1978, 1980; Watson and Tellegen, 1985). Russell (1980) proposed a circumplex model of affect (referred to herein as the UCMA) in which discrete affective states could be modeled as combinations of two affective dimensions, which he labeled *valence*, ranging from good (i.e., pleasure) to bad (i.e., displeasure), and *arousal*, ranging from high (i.e., alert) to low (i.e., sleepy). Watson and Tellegen (1985) later proposed a modified circumplex model emphasizing affective dimensions that were rotated 45 degrees (around the circumplex; i.e., RCMA) from Russell (1980) original model. They labeled the dimensions PA, ranging from the union of positive valence and high activation (e.g., excited) to the union of negative valence and low activation (e.g., sluggish), and NA, ranging from the union of negative valence and high activation (e.g., distressed) to the union of negative positive and low activation (e.g., relaxed).

**Figure 1:**
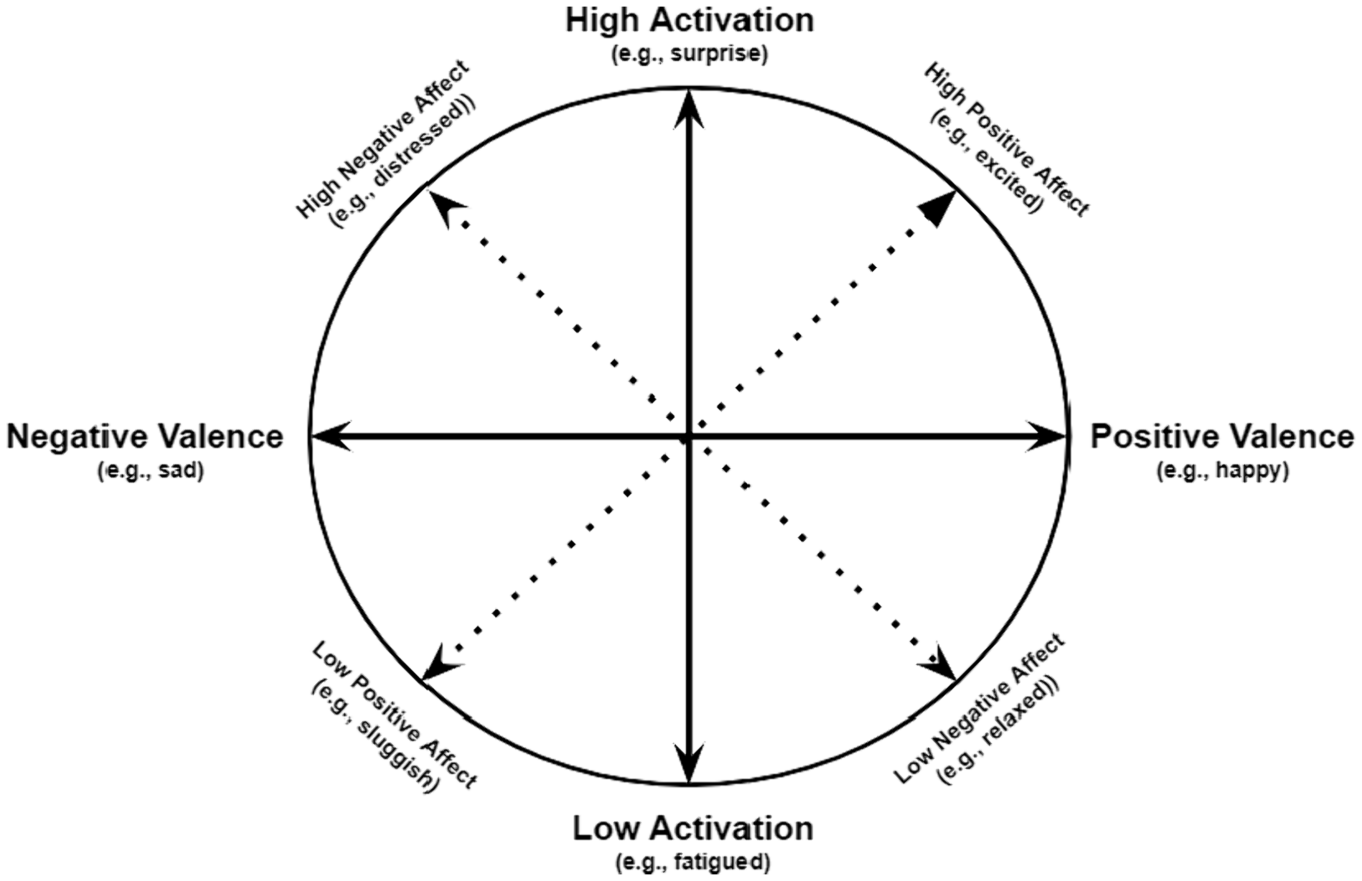
Comparison of unrotated (UCMA) versus rotated (RCMA) circumplex models of affect. The solid arrow lines represent dimensions of the UCMA, and the dotted lines represent the dimensions of the RCMA.

Importantly, the two circumplex models posited by Russell (1980) and Watson and Tellegen (1985) are complementary rather than competing (see Watson and Tellegen, 1985, p. 221–222; Watson et al., 1999, p. 821, 828). Both posit the same basic affective structure situated around a two-dimensional circular space. Where they differ is in the rotation and labeling of the two affective dimensions. Figure 1 illustrates the correspondence of the UCMA and RCMA in the same two-dimensional circumplex, but with differences in the labeling and orientation of the two affective dimensions (for similar diagrams comparing the UCMA and RCMA see Yik et al., 1999; Ekkekakis, 2013).

As shown in Figure 1, the valence and activation dimensions of the UCMA have a 90-degree separation and are thus posited to be orthogonal. Likewise, the PA and NA dimensions of the RCMA also have a 90-degree separation and are thus posited to be orthogonal. The two circumplex models differ regarding their emphasis on valence and arousal versus PA and NA, but they describe the same two-dimensional affective space. Moreover, the PA and NA dimensions of the RCMA are rotated 45 degrees from the valence and activation dimensions of the UCMA. Thus, while feeling “happy” is an example of pure positive valence, it is not, according to the RCMA, an example of PA, as the latter includes a combination of positive valence and high arousal, with “excited” as the prototypical example. Likewise, while feeling “sad” is an example of pure negative valence, it is not, according to the RCMA, an example of NA, because it is spaced 45 degrees from feeling distressed, which is the prototypical example of pure NA.

In the UCMA, each of the two affective dimensions are bipolar. The valence dimension ranges from extreme pleasure at one pole to extreme displeasure at the opposite pole. The activation dimension ranges from extreme high activation (e.g., extremely alert) at one pole to extreme low activation (e.g., extremely sleepy) at the opposite pole. Importantly, the two affective dimensions in the RCMA are also posited to be bipolar. The PA dimension ranges from extreme positive valence combined with high activation (e.g., excited) at one pole to extreme negative valence combined with low activation (e.g., sluggish) at the opposite pole. The NA dimension ranges from extreme negative valence combined with high activation (e.g., distressed) at one pole to extreme positive valence combined with low activation (e.g., relaxed) at the opposite pole.

However, whereas the UCMA places equal emphasis on the full range of affect across both valence and arousal dimensions, the RCMA places greater emphasis on the high arousal poles of the Positive Activation and Negative Activation dimensions—that is, the upper half of the circumplex in Figure 1. This emphasis on high (rather than low) activation states in the RCMA reflects the authors’ position that affect is defined in terms of high activation states, as illustrated in the following quotations from the authors of the RCMA:

Specifically, in our own studies and in virtually all published self-report studies that we have subsequently reanalyzed, we have encountered the same two large, bipolar dimensions: Positive Affect and Negative Affect… These two factors have been characterized as *“descriptively bipolar* but *affectively unipolar* dimensions” (Zevon and Tellegen, 1982, p. 112) to emphasize that only the high end of each dimension represents a state of emotional arousal (or high affect), whereas the low end of each factor is most clearly and strongly defined by terms reflecting a relative absence of affective involvement (e.g., *calm* and *relaxed* for Negative Affect, *dull* and *sluggish* for Positive Affect) (Watson and Tellegen, 1985, p. 220–221).

… as our view of these dimensions has evolved, we increasingly have come to see them as truly unipolar constructs that essentially are defined by their high poles. As we discuss in greater detail shortly, we now view these dimensions as reflecting two basic biobehavioral systems of activation. As such, the activated, high ends of the dimensions fully capture their essential qualities. Moreover, although terms such as *sluggish* and *relaxed* can be used to characterize low levels on these dimensions in certain contexts, they do not intrinsically define the dimensions themselves. This is because the low poles of these dimensions ultimately reflect the absence of a particular kind of activation rather than the presence of a certain affective state (such as sluggishness or relaxation) (Watson et al., 1999, p. 827).

In sum, while the original RCMA included two bipolar dimensions of PA and NA, the emphasis in the model is clearly on the high-activation poles of the two dimensions, with more recent writings from the authors indicating a largely unipolar description of the PA and NA dimensions.

## The “received view” of the RCMA

There has been pervasive misunderstanding of the RCMA, leading to misinterpretation of empirical findings and hindering scientific progress in affective science. Specifically, many researchers (see empirical review below) have erroneously interpreted the RCMA to say that the PA dimension includes all positively valenced states and the NA dimension includes all negatively valenced states. However, as illustrated in the following quotation from the authors of the RCMA, this is not the case:

… although the name *Positive Affect* implies that it is a dimension reflecting fluctuations in positively valenced mood states, Figure 1 indicates that it actually contains unpleasant, negatively valenced terms (e.g., *dull, sluggish*) at its low pole. In parallel fashion, the *Negative Affect* dimension—which should tap variations in negatively valenced states—actually includes pleasant, positively valenced affects (e.g., *relaxed, calm*) at its low end (Watson et al., 1999, p. 826).

Indeed, according to the Figure 1 referred to in the above quotation (also see Figure 1 in the present paper), in the RCMA, the affective states sluggish, drowsy, dull, sleepy, and blue, grouchy, lonely, sad, unhappy are not examples of NA. Instead, feeling sluggish, drowsy, dull, or sleepy represents low PA according to the RCMA, whereas feeling blue, grouchy, lonely, sad, or unhappy represents the negative pole of the valence dimension of the UCMA. Likewise, the affective states relaxed, restful, calm, placid, and content, happy, pleased, satisfied are not, according to the RCMA, examples of PA. Instead, feeling relaxed, restful, calm, placid represent low NA according to the RCMA, whereas feeling content, happy, pleased, or satisfied represents the positive pole of the valence dimension of the UCMA (Figure 1). This may come as a surprise to many researchers who assume that sadness is, according to the RCMA, an example of NA, or that happiness is an example of PA.

Unfortunately, the labels PA and NA as used in the RCMA led to considerable confusion in the literature, as was subsequently acknowledged by the authors of the RCMA:

… the terms “positive affect” and “negative affect” have been used inconsistently by different writers. In early studies of self-rated affect, researchers tended to use the terms indiscriminately to refer to any positively and negatively valenced feeling states (for a discussion, see Watson and Tellegen, 1985). This produced widespread confusion in the literature, because—as we noted earlier—different types of mood descriptors actually show substantially different intercorrelations (Watson and Tellegen, 1999, p. 602)… The literature is so confused at this point that the terms “positive affect” and “negative affect” perhaps should indeed be used only as inclusive terms referring to any positive and negative feeling states (Watson and Tellegen, 1999, p. 602–603).

Indeed, in a separate paper, the authors of the RCMA admitted that the PA and NA labels may have been a major source of confusion and suggested that, as a potential remedy, the dimensions of the RCMA be changed from Positive Affect and Negative Affect to Positive Activation and Negative Activation:

Are the labels *Negative Affect* and *Positive Affect,* in fact, misleading? In our view, the most serious criticism of these names is that they misrepresent the actual valence of these dimensions (see Feldman Barrett and Russell, 1998)… Although this objection has some merit, we have not found it persuasive… Nevertheless, it also has become obvious that these terms [Positive Affect and Negative Affect] fail to convey the activated nature of these dimensions adequately. Therefore, in light of the evidence we consider later, we believe that the labels *Negative Activation* (or *NA*) and *Positive Activation* (or *PA*) provide a better, more accurate representation of these dimensions” (Watson et al., 1999, p. 826–827).

This was a welcome change. Whereas the original labels “Positive Affect” and “Negative Affect” are easily misinterpreted to refer to positive valence and negative valence, respectively, the labels “Positive Activation” and “Negative Activation” clarify that the PA dimension is a union of positive valence and high activation and the NA dimension is a union of negative valence and high activation. Unfortunately, few researchers have adopted the revised labels of “Positive Activation” and “Negative Activation” (see review below). Thus, the confusion in the literature regarding the meaning of the labels “Positive Affect” and “Negative Affect” persists.

In the present paper, we retain the abbreviations PA and NA, with acknowledgment that they refer to the two dimensions of the RCMA, regardless of whether those dimensions are labeled “Positive Affect” and “Negative Affect” (i.e., Watson and Tellegen, 1985) or “Positive Activation” and “Negative Activation” (Watson et al., 1999).

A second misreading of the RCMA—that stems directly from the one previously discussed—is that positively valenced states (e.g., pleasure, happy) and negatively valenced states (e.g., displeasure, sad) are orthogonal and uncorrelated. This misunderstanding presumably occurs because having first erroneously interpreted PA as encompassing all positively valenced states and NA as encompassing all negatively valenced states, researchers then correctly read the RCMA to say that PA and NA are orthogonal. Taken together, this leads to an erroneous reading of the RCMA in which *positively valenced states* and *negatively valenced states*, rather than PA and NA, are orthogonal and thus should be empirically uncorrelated.

Indeed, in their foundational paper on the RCMA, Watson and Tellegen (1985) state that PA and NA are not opposites, but instead independent, uncorrelated dimensions: “Although the terms Positive Affect and Negative Affect might suggest to some readers that these mood factors are opposites (i.e., negatively correlated), they are in fact independent, uncorrelated dimensions…” (p. 221). If one has already erroneously interpreted the RCMA as saying that PA includes all positively valenced states and NA includes all negatively valenced states, then this statement will likely be interpreted to mean that positively and negatively valenced states are orthogonal.

However, it is critically important to interpret the prior quotation in the context of the specialized meaning of PA and NA in the RCMA. In the context of the RCMA, the labels “Positive Affect” and “Negative Affect” refer to the rotated affective dimensions exemplified by feelings of excitement and distress, respectively; not to positively valenced states (e.g., pleasure, happy) and negatively valenced states (e.g., displeasure, sad). Thus, Watson and Tellegen (1985) statement that PA and NA are orthogonal means that feelings of excitement and distress are orthogonal—not feelings of pleasure/happiness and displeasure/sadness.

In fact, according to the RCMA, pleasure/happiness and displeasure/sadness represent opposite (not independent) poles of the same bipolar affective dimension and thus should be empirically negatively correlated. This is illustrated in the following quotations from the authors of the RCMA:

“Most notably, we agree that self-rated affect is characterized by a bipolar dimension of pleasant versus unpleasant feeling (indeed, as we discuss subsequently, this point has not provoked any significant controversy in the affect literature)…

From the beginning, we have carefully distinguished between two types of positively and negatively valenced terms that correlate quite differently with one another. On the one hand, markers of what we have called “high positive affect” (e.g., *active, enthusiastic, interested*) and “high negative affect” (e.g., *afraid, angry, guilty*) tend to be only weakly related to one another, at least when raw, uncorrected correlational data are obtained (a point we explore subsequently). On the other hand, indicators of “pleasantness” (e.g., *happy, content, pleased*) and “unpleasantness” (e.g., *unhappy, sad, blue*) consistently show moderate to strong negative correlations, even with uncorrected data…

Tellegen et al. (1994) pointed out that although terms such as *happy* and *sad* are at opposite ends of the bipolar pleasantness versus unpleasantness dimension in Watson and Tellegen’s model, “this bipolarity does not characterize all opposite-valenced pairs” (p. 5); as an example of nonbipolarity, they noted that although terms such as *enthusiastic* and *distressed* are also opposite in valence, they are located on the independent “activation axes” of positive and negative affect in Watson and Tellegen’s map…

In this more precise terminological scheme, markers of positive affect and negative affect should consistently show weak negative correlations, whereas terms reflecting pleasantness and unpleasantness should tend to be strongly negatively correlated (and, hence, define a single bipolar dimension)” (Watson and Tellegen, 1999, p. 601–603).

“We must emphasize that certain portions of the affect circumplex (such as the existence of a bipolar valence dimension similar to our higher order happiness vs. unhappiness factor, as well as separate positive and negative activation dimensions) are well established and clearly reflect robust properties of self-rated affect. Nevertheless, as researchers who have articulated a very similar circular scheme (Watson and Tellegen, 1985), we believe it is time to move beyond these well-known structures.

We recently proposed a three-level hierarchical structure that better captures the complexities of affective experience (see Tellegen et al., in press). A general bipolar dimension of happy versus unhappy feeling states emerges at the apex of this hierarchy, attesting to its pervasiveness in self-rated affect” (Watson and Tellegen, 1999, p. 609).

“As discussed previously, we believe that the bipolar Pleasantness-Unpleasantness dimension reflects important and intrinsic qualities of affective experience; as such, it is essential to any complete understanding of mood” (Watson et al., 1999, p. 829).

“Thus, Figure 1 places moderate-activation variables, such as “happy” and “sad,” at opposite poles of the same dimension: Pleasantness-Versus-Unpleasantness; but it assigns high-activation variables, such as “enthusiastic” and “distressed,” to different and relatively independent dimensions: PA and NA, respectively.” (Tellegen et al., 1999, p. 298).

From these quotations it is clear that, consistent with the UCMA, the authors of the RCMA posit that pleasure/happiness and displeasure/sadness represent opposite (not independent) poles of the same bipolar affective dimension. Unfortunately, the misinterpretation of the labels PA and NA has set up an apparent, but artificial, distinction between the UCMA and the RCMA in which the UCMA holds that feeling happy/good/pleasant is the opposite of feeling sad/bad/unpleasant but the RCMA holds that they are independent and thus should be unrelated:

… some researchers have created a false conflict between what typically are called the “bipolarity” and “independence” models of affective structure (see, e.g., Green et al., 1993). In brief, the independence view—which usually is attributed to researchers such as Bradburn (1969) and ourselves—is defined as positing the existence of orthogonal dimensions of positive and negative affect. In contrast, the bipolarity view—attributed to Russell and his colleagues—is characterized as strongly asserting the existence of a bipolar valence dimension of positive versus negative feeling. These two models are further characterized as mutually incompatible and in conflict with one another. This, in turn, suggests that there is some fundamental incompatibility between the two-factor structure of Watson and Tellegen (1985) and the circumplex model articulated by Russell and his colleagues (e.g., Russell, 1980; Feldman Barrett and Russell, 1998; Russell and Carroll, 1999a,b; Watson and Tellegen, 1999, p. 603).

“If proponents of independence can simultaneously argue for the existence of a bipolar valence dimension—and, more fundamentally, if theorists such as Russell, Larsen and Diener, and ourselves all have espoused very similar views regarding bipolarity and independence—then the reader may well wonder, “What exactly is the nature of this ongoing controversy?” In this regard, we agree with Russell and Carroll (1999a,b) that to a considerable extent, this apparent conflict actually is a pseudocontroversy that emerged because of continuing sources of confusion in the field” (Watson and Tellegen, 1999, p. 602).

As can be seen from these quotations, both the UCMA and the RCMA posit that feeling happy/good/pleasant is the opposite of feeling sad/bad/unpleasant, and thus measures of these affective states should be negatively correlated. Accordingly, neither the UCMA nor the RCMA posit that feeling happy/good/pleasant is orthogonal to feeling sad/bad/unpleasant and thus, neither posit that measures of these affective states should be uncorrelated.

## Misinterpretations of the PANAS

The PANAS was created by the authors of the RCMA as a tool for assessing self-reported PA and NA. In the original publication of the PANAS, the authors again clarified the specific meaning of PA and NA:

Although the terms *Positive Affect* and *Negative Affect* might suggest that these two mood factors are opposites (that is, strongly negatively correlated), they have in fact emerged as highly distinctive dimensions that can be meaningfully represented as orthogonal dimensions in factor analytic studies of affect. Briefly, Positive Affect (PA) reflects the extent to which a person feels enthusiastic, active, and alert. High PA is a state of high energy, full concentration, and pleasurable engagement, whereas low PA is characterized by sadness and lethargy. In contrast, Negative Affect (NA) is a general dimension of subjective distress and unpleasurable engagement that subsumes a variety of aversive mood states, including anger, contempt, disgust, guilt, fear, and nervousness, with low NA being a state of calmness and serenity” (Watson et al., 1988, p. 1063).

Consistent with their other writings on the RCMA, in the above passage, Watson et al. (1988) clarify that (a) PA does not encompass all positively valenced states and NA does not encompass all negatively valenced states, and (b) PA and NA are orthogonal dimensions of affect—not opposites. That is, according to the RCMA, PA is not synonymous with feeling good, happy, or pleasure, and NA is not synonymous with feeling bad, sad, or displeasure.

Moreover, in developing the PANAS, Watson et al. (1988) took care to ensure that when using the measure, PA and NA would be empirically uncorrelated, consistent with their theoretical independence:

Our greatest concern was to select terms that were relatively pure markers of either PA or NA; that is, terms that had a substantial loading on one factor but a near-zero loading on the other… we selected those terms that had an average loading of 0.40 or greater on the relevant factor… Twenty PA markers and 30 NA markers met this initial criterion. However, as noted previously, we were also concerned that the terms not have strong secondary loadings on the other factor. We therefore specified that a term could not have a secondary loading of |0.25| or greater… This reduced the pool of candidate descriptors to 12 for PA and 25 for NA” (Watson et al., 1988, p. 1064).

Unfortunately, many authors have cited the absence of significant correlations between the PA and NA scales of the PANAS as evidence of the orthogonality of positively and negatively valenced states. This, however, is a misinterpretation of the PANAS and the RCMA that undergirds it. The PA and NA scales of the PANAS reflect the rotated PA and NA dimensions of the circumplex model, not, as is often thought, positive valence and negative valence. Moreover, as noted above, the PA and NA scales of the PANAS were intentionally designed to be uncorrelated (consistent with the RCMA) such that any instance of a correlation between them would be a statistical anomaly. Therefore, it is not even possible to use the PANAS to answer questions about the orthogonality of positively and negatively valenced states.

## Review of research demonstrating the “received view”

In this section we examine the prevalence of the “received view” of the RCMA that positively valenced states and negatively valenced states are orthogonal, using a random selection of empirical research on affect published in high-impact psychology journals that are likely to have the widest readership and, thus, the biggest impact on the field. We first examined the proportion of papers that subscribed to the RCMA—as evidenced by either citing an RCMA foundational paper or using the PANAS or a PANAS-derived measure—and therefore inherently adopted the RCMA principle that PA and NA are orthogonal. We then examined the proportion of RCMA-subscribing articles that had evidence of the “received view” of the RCMA. A secondary aim was to examine predictors of the “received view,” such as recency of publication, journal specialization (affect/emotion versus other), and citation of foundational RCMA papers. Specifically, we hypothesized that more recent publications, those in affect/emotion journals, and those citing at least one of the foundational papers on the RCMA (quoted above) would have a lower proportion of articles supporting the “received view” of the RCMA.

## Transparency and openness

The present review is a systematic summary of authors’ interpretations of theoretical principles and empirical findings; however, it is not a traditional systematic review in which empirical findings are summarized. While we were not able to locate a reporting guideline that fit well with the goals of the review, wherever possible, we include details consistent with the PRISMA 2020 guidelines for systematic reviews (Page et al., 2021). This review was not preregistered.

## Methods

### Journal and article selection

We reviewed a random sample of 140 journal articles reporting empirical research on affect that were published since 2000 in 14 selected psychology journals chosen for their high impact factors and coverage of a range of psychological subdisciplines (see Table 2). Specifically, we selected the two highest-impact journals (based on Journal Citation Reports, 2021) in seven psychological subdisciplines (i.e., general, clinical, industrial/organizational, child/developmental, social, medical/health, affect/emotion). Within each journal, we used PsycInfo to search for empirical research articles published during or after 2000, that included the terms “positive affect,” “negative affect,” “positive activation,” “negative activation,” “positive activated affect,” or negative activated affect” in the title or abstract. Journals that had fewer than 20 articles meeting these search criteria were excluded and replaced by the next highest-impact journal in that subdiscipline. The literature search yielded 922 potentially relevant records across the top 14 journals. We then randomly selected 10 articles from each journal to be included in the review. Full texts of the selected articles were reviewed, and those that did not use any of the search terms (i.e., “positive affect,” “negative affect,” “positive activation,” “negative activation,” “positive activated affect,” or negative activated affect”) in the manuscript itself (i.e., search terms were only included in either the title or abstract) were excluded (*n* = 5). The final sample consisted of 135 articles. Supplementary Appendix A contains a reference list of all included studies. Supplementary Appendix B contains extracted data from all articles.

**Table 2:** Description and examples of coding domains.

Coding domain	Description	Example
**Evidence of subscribing to the RCMA**		
Explicit statement that the RCMA is the primary model of affect	Included statement(s) indicating that Watson’s circumplex model (which we term the RCMA) informed the study design and/or interpretation	“Items assessing positive affect were based on the circumplex model (i.e., valence and arousal) of the Positive and Negative Affect Schedule (Watson et al., 1988) with response options on a 4-point Likert scale ranging from 1 (*not at all*) to 4 (*extremely*)” (Maher et al., 2018).
Cited Watson in related to the measurement and/or conceptualization of affect	Included reference(s) to published work by Watson that was used to support the authors’ conceptualization of affect	“Finally, we make a case that because both PA and NA are activated states (Watson et al., 1988), they should exhibit similar relationships with heart rate (a physiological indicator of activation; Kamarck et al., 2005) at the within-individual level” (Ilies et al., 2010).
Used an established PANAS measure	Used an established PANAS measure, including both the original PANAS and alternative PANAS versions, such as the PANAS-X, PANAS-SF, or I-PANAS-SF	“The pre-and post-task positive and negative affects of participants were measured with the Positive and Negative Affect Schedule (Watson et al., 1988), a 20-item measure of an individual’s experienced positive (e.g., proud, excited) and negative (e.g., upset, distressed) affective states” (Erez et al., 2008).
Used a novel PANAS-based measure	Used any measure of affect that the authors indicate was derived from or based on an established PANAS measure	“The positive affect measure was based on the widely used positive and negative affect schedule (PANAS; Watson et al., 1988)…” (Curhan et al., 2014)
**Evidence of view that PA = positive valence and NA = negative valence**		
Included low or neutral positively valenced states in the measure of PA	Included either low (e.g., calm) or neutral (e.g., pleasure) activation, positively valenced states in the measure of PA	“Nine PA words (happy, excited, cheerful, pleasant, calm, energetic, enthusiastic, content, and relaxed) were averaged to create a daily PA value (Cronbach’s α range for each of the 13 days = 0.88–0.92)” (Jenkins et al., 2020).
Included low or neutral negatively valenced states in the measure of NA	Included either low (e.g., depressed) or neutral (e.g., displeasure) activation, negatively valenced states in the measure of NA	“A negative affect (NA) measure was created by averaging the scores for each of the following items (i.e., emotionally upset, stressed, lonely/ alone, annoyed/angry, tense/anxious, sad/depressed, and discouraged/frustrated; Chronbach’s *α* = 0.851)” (Dunton et al., 2009).
Included low or neutral positive activation states in the description of PA or interpretation of results	Included either low (e.g., calm) or neutral (e.g., pleasure) activation, positively valenced states in the description/conceptualization of PA or interpretation of results (e.g., scores on a measure of PA)	“For example, it is unclear how the various facets of hedonic functioning relate to positive affect (Treadway and Zald, 2011), which has long been considered a fundamental dimension of pleasure and joy” (Kovacs et al., 2016).
Included low or neutral negative activation states in the description of NA or interpretation of scores on a measure of NA	Included either low (e.g., depressed) or neutral (e.g., displeasure) activation, negatively valenced states in the description/conceptualization of NA or interpretation of results (e.g., scores on a measure of NA)	“Negative affect (NA) refers to the subjective experience of an array of negative emotions (e.g., fear, anger, sadness, guilt)…” (Naragon-Gainey and DeMarree, 2017)

### Extraction, coding, and analysis

First, we used NVivo (QSR International Pty Ltd., 2022) to review and extract relevant details about each study, including (1) definition(s) of affect, PA, and/or NA, (2) measure(s) of affect (e.g., PANAS), (3) statement(s) that made reference to foundational papers on affect by Watson and/or Russell, (4) statement(s) that included the word “circumplex,” and (5) statement(s) that included the term(s) “affect,” “PA,” “NA,” “feeling,” and/or “valence.” Statements were extracted at the sentence-level (see Supplementary Appendix B for details regarding extraction guidelines). Second, two coders (MASKED FOR REVIEW) independently reviewed and coded the extracted statements (as well as Supplemental material if/when it was referenced in the extracted text) using a data coding template (Covidence Systematic Review Software, 2024). The data coding template was developed to assess (1) evidence of subscribing to the RCMA, (2) evidence of the view that PA includes all positively valenced states and NA includes all negatively valenced states (in combination, (1) and (2) equate to the “received view”), and (3) predictors of the “received view” (see Supplementary Appendix C). Discrepancies were discussed and resolved by a third investigator (MASKED FOR REVIEW).

#### Evidence of subscribing to the RCMA

To assess whether papers had evidence of subscribing to the RCMA, we coded information related to whether the paper: (1) explicitly stated that the RCMA was their primary model of affect (i.e., the model of affect that was used to guide conceptualization of the study/findings), (2) cited Watson in relation to the conceptualization of affect (i.e., the reference to Watson was used to guide conceptualization of the study/findings), and (3) used an established PANAS measure (including both the original PANAS and alternative PANAS versions, such as PANAS-X, PANAS-SF, I-PANAS-SF) or a novel PANAS-based measure (i.e., any measure of affect that the authors indicate was derived from or based on an established PANAS measure). The latter criterion was retained with acknowledgement that many researchers may use the PANAS without fully appreciating its theoretical underpinnings. Nonetheless, because the construction of the PANAS measures is grounded in the RCMA, it necessitates adoption of the conceptual underpinnings of the RCMA for accurate interpretation of the findings that stem from PANAS measures. Thus, we included use of the PANAS as a criterion for subscription to the RCMA while acknowledging that such subscription may be implicit.

See Table 3 for additional description of coding criteria as well as example statements. We then computed a variable reflecting whether papers had evidence of subscribing to the RCMA (i.e., met at least one of the above criteria).

**Table 3:** Journals included in review.

Domain	Journal	2019 Impact factor
General	Psychological Science	5.367
	British Journal of Psychology	3.239
Clinical	Journal of Abnormal Behavior	6.484
	Clinical Psychological Science	5.415
Industrial/Organizational	Journal of Applied Psychology	5.818
	Journal of Occupational Health Psychology	7.329
Child/Development	Journal of Child Psychology and Psychiatry	7.035
	Child Development	4.891
Social	Journal of Personality and Social Psychology	6.315
	Social Psychological and Personality Science	4.380
Medical/Health	Psychological Medicine	5.811
	Annals of Behavioral Medicine	4.475
Affect/Emotion	Journal of Positive Psychology	3.819
	Emotion	3.177

#### Evidence of view that PA = positive valence and NA = negative valence

According to the RCMA, high PA is defined as the intersection of positive valence and high arousal (e.g., excitement) and high NA is defined as the intersection of negative valence and high arousal (e.g., distress). However, in the “received view” of the RCMA, PA includes all positively valenced states, not just those that are high in arousal, and NA includes all negatively valenced states, not just those that are high in arousal. Thus, to assess whether papers had evidence of the view that PA includes all positively valenced states and NA includes all negatively valenced states, we coded information related to whether the paper: (1) included either positively valenced but low (e.g., calm) or neutral (e.g., pleasure) activation states in the measure of PA, and/or negatively valenced but low (e.g., depressed) or neutral (e.g., displeasure) activation states in the measure of NA, and (2) included either low or neutral activation states in the operational description of PA or NA or interpretation of study findings (Table 3) or NA. We computed a variable to reflect whether papers had evidence of viewing PA as all positively valenced states and NA as all negatively valenced states (i.e., did not limit the measure/description to high activation states).

#### Prevalence of the “received view”

We determined the proportion of papers that had evidence of viewing PA as all positively valenced states and/or NA as all negatively valenced states from among those papers subscribing to the RCMA. This provided an indication of the prevalence of the “received view” of the RCMA that positively and negatively valenced affective states (rather than PA and NA) are theoretically orthogonal. We used the number of papers showing evidence of subscription to the RCMA as the denominator because it is not necessarily theoretically inconsistent to define PA and NA in terms of valence—rather than as defined by the RCMA—if one is not subscribing to the RCMA.

#### Predictors of the “received view”

To assess additional hypothesized predictors of the “received view,” we extracted the journal title and year of publication (which was then categorized into 5-year periods plus the most recent 2-year period: 2000–2004, 2005–2009, 2010–2014, 2015–2019, 2020–2021). We used chi-square tests of independence to determine whether the proportion of papers that had evidence of the “received view” differed by (1) journal topic (i.e., affect/emotion journal vs. other journal), (2) whether or not Watson was cited in support of affect conceptualization (yes vs. no), and (3) year of publication (2000–2004 vs. 2005–2009 vs. 2010–2014 vs. 2015–2019 vs. 2020–2021).

The literature search was conducted in March 2021, with extraction and coding conducted between August 2021 and August 2022, and data analysis from August to September 2022.

## Results

### Evidence of use of the RCMA

A total of 135 studies were included in the review. Overall, we found evidence that 42.2% of papers (*n* = 57) subscribed to the RCMA. Specifically, although only 1 paper (0.7%) explicitly referenced the RCMA as being the guiding theory of affect, one-fifth (*n* = 27) of the papers reviewed cited Watson in relation to the conceptualization of affect, and more than one-third (*n* = 47, 34.8%) of papers used a PANAS or PANAS-based measure of affect.

### Evidence of view that PA = positive valence and NA = negative valence

In over half of the papers (*n* = 69, 51.1%), we found evidence of the view that PA includes all positively valenced states (including low/neutral activation states) and/or that NA includes all negatively valenced states (including low/neutral activation states). Specifically, almost one-fifth (*n* = 26, 19.3%) of papers described PA as including low/neutral activation positively valenced states (e.g., calm, content) and/or NA as including low/neutral activation negatively valenced states (e.g., sad, depressed), and almost half (*n* = 54, 40%) of papers included either low/neutral activation positively valenced states in their measure of PA, and/or low/neutral negatively valenced states in their measure and/or conceptualization of NA. Additional descriptive information is presented in Table 4.

**Table 4:** Descriptive information.

	Among all papers (*N* = 135) *n* (%)
Evidence of Using RCMA	57 (42.2%)
**Primary model of affect (explicitly referred to)**	
RCMA*	1 (0.7%)
UCMA	6 (4.4%)
**Authors cited in support of affect conceptualization**	
Watson*	27 (20.0%)
Russell	8 (5.9%)
**Measure of affect**	
PANAS*	16 (11.9%)
PANAS-SF*	2 (1.5%)
I-PANAS-SF*	2 (1.5%)
Modified PANAS*	28 (20.7%)
Other measure of affect	71 (52.6%)
**Evidence of view that PA = positive valence and NA = Negative valence**^┼^	69 (51.1%)
Measure of affect did not limit PA/NA to high activation states	54 (40.0%)
Description of affect did not limit PA/NA to high activation states	26 (19.3%)

*Rows contribute to the total number of papers showing evidence of use of RCMA. ^**┼**^Excludes overlap between measure/description of affect.

### Prevalence and predictors of the “received view”

Among the 57 papers that used the RCMA, more than half (52.6%, *n* = 30) had evidence of the “received view” (see Table 4). Predictors of the “received view” were examined from among the 57 papers employing the RCMA (see Table 5). Papers that cited Watson in reference to the conceptualization of affect were more likely to have evidence of the “received view” (χ^2^ = 6.48; *p* = 0.01). Specifically, 70.4% of papers citing Watson had evidence of the “received view,” versus 36.7% of those who did not cite Watson. There was no difference in prevalence of the “received view” among articles published in an affect/emotion journal (χ^2^ = 0.02; *p* = 0.89). Specifically, 50% of papers published in affect/emotion journals had evidence of the “received view,” versus 52.9% of those published in other journals. There was also no association between year of publication and prevalence of the “received view” (χ^2^ = 6.05; *p* = 0.20).

**Table 5:** Prevalence of the “received view” of the rotated circumplex model of affect (RCMA).

Total papers in each category	Evidence of “received view” of the RCMA* n (%)
Evidence of using RCMA (*N* = 57)	30 (52.6%)
**Reference to Watson**	
Yes (*n* = 27)	19 (70.4%)
No (*n* = 30)	11 (36.7%)
**Journal**	
Affect/Emotion journal (*n* = 6)	3 (50.0%)
Other journal (*n* = 51)	27 (52.9%)
**Year**	
2000–2004 (*n* = 4)	2 (50%)
2005–2009 (*n* = 6)	4 (66.7%)
2010–2014 (*n* = 13)	4 (51.2%)
2015–2019 (*n* = 24)	12 (50%)
2020-present (*n* = 10)	8 (80%)

*Frequencies and proportions in this column reflect the volume of papers subscribing to the erroneous “received view” that (a) PA includes all positively valenced states and NA includes all negatively valenced states (this is inconsistent with the RCMA), and (b) PA and NA are orthogonal (this is consistent with the RCMA). Taken together, this amounts to the proposition—inconsistent with the RCMA—that positively valenced states (e.g., feeling good, pleasure) and negatively valenced states (e.g., feeling bad, displeasure) are necessarily orthogonal.

### *Post hoc* analyses

The RCMA posits that the PA dimension is a union of positive valence and high activation and the NA dimension is a union of negative valence and high activation, thus PA and NA should be limited to high activation states and exclude both neutral and low activation states. However, given that there is not clear consensus regarding the threshold between neutral and high activation states, we conducted post-hoc analyses to examine the prevalence of the “received view” when only low activation states were coded as being inconsistent with the RCMA. In other words, in the post-hoc analysis only papers that included either low activation positively valenced states in the measure/description of PA or low activation negatively valenced states in the measure/description of NA were considered evidence that PA includes all positively valenced states and NA includes all negatively valenced states. Across all papers (*N* = 135), nearly one-third (*n* = 42, 31.1%) included low activation positively valenced states in either their measure and/or conceptualization of PA, and/or low activation negatively valenced states in their measure and/or conceptualization of NA. Among the 57 papers that had evidence of subscribing to the RCMA, 29.8% (*n* = 17) had evidence of the more restricted “received view” that all positively valenced states (including low activation states such as calm) and negatively valenced states (including low activation states such as depressed) are orthogonal.

## Discussion

The misinterpretation of “positive affect” and “negative affect” as conceptualized by the RCMA has been well-documented (e.g., Russell and Barrett, 1999; Russell and Carroll, 1999a,b; Tellegen et al., 1999; Watson et al., 1999; Watson and Tellegen, 1999; Yik et al., 1999; Ekkekakis and Petruzzello, 2002; Ekkekakis, 2008; Ekkekakis and Zenko, 2016); however, this is the first study to provide evidence of its pervasiveness throughout the field of psychology. Specifically, we conducted a review of high-impact research across seven psychological disciplines to determine the prevalence of the “received view” of the RCMA that positively valenced states (e.g., feeling good, pleasure, happy) and negatively valenced states (e.g., feeling bad, displeasure, sad)—rather than PA and NA, as defined by the RCMA—are theoretically orthogonal. Results indicated that more than half of the papers that were included in this review, and used the RCMA, had evidence of the “received view,” demonstrating that misuse of the terms PA and NA is rampant in the psychological literature.

Papers that cited Watson in relation to the conceptualization of affect were significantly more likely to have evidence of the “received view,” which is particularly notable given that Watson himself has emphasized that the terms PA and NA should be reserved for high activation states (i.e., the orthogonal dimensions mapped in Figure 1). Type of journal (i.e., affect/emotion journal vs. other journals) and year of publication did not influence prevalence of the “received view,” suggesting that there is not greater discretion regarding this theoretical inconsistency among reviewers and editors of affect/emotion journals nor has there been a reduction in misinterpretation of the RCMA over time.

The misunderstandings of the RCMA, as well as corresponding misinterpretations of the PANAS, have led to widespread confusion in the field regarding the nature of affect. Most prominently, the “received view” has led many authors to incorrectly conclude that all forms of positive valence (e.g., happiness) and negative valence (e.g., sadness)—rather than PA and NA, as defined by the RCMA—are theoretically orthogonal. Because of the impact of the RCMA and the PANAS on the field of psychology, the “received view” of the RCMA, as well as its downstream implications regarding the nature of affect permeates academic psychology. Although Watson has since tried to correct confusion surrounding the misinterpretation of PA and NA by recommending use of the terms “positive activation” and “negative activation” (Watson et al., 1999), none of the papers included in this review adopted that language.

While peripheral to our primary aims, it should be noted that over half of the papers included in this review defined PA and NA as including all positively/negatively valenced states—a definition that is inconsistent with the RCMA. While we subsequently focused our analyses on what we have termed here the “received view” (i.e., the subset of papers for which there was direct evidence of subscription to the RCMA *and* evidence that PA and NA are defined as including all positively/negatively valenced states), it is also noteworthy that there is a large proportion of papers for which PA and NA are defined as including all positively and negatively valenced states, even if there was no direct evidence of use of the RCMA. The latter suggests that the terms PA and NA—which are strongly associated with the RCMA—are typically not defined in a way that is consistent with the RCMA, regardless of whether the authors appear to be directly subscribing to the RCMA. For papers that do not indicate subscription to the RCMA, it is not necessarily inconsistent to define PA and NA in terms of valence. Nonetheless, use of PA and NA in ways that deviate from their RCMA-based definition is likely to add further confusion in the literature.

In addition to the theoretical implications, the pervasiveness of the “received view” may lead to inappropriate applications of research findings to clinical treatments. For example, relaxation training has been shown to reduce anxiety (Francesco et al., 2010). However, if some researchers believe that greater relaxation is indicative of decreased NA (consistent with the RCMA), while others believe that it is indicative of increased PA (consistent with the “received view”), this may lead to disagreement regarding the underlying mechanism (e.g., NA vs. PA) responsible for observed treatment effects. Lack of clarity surrounding the mechanism of action may then prohibit researchers from identifying additional intervention components that will target the desired mechanism and further improve treatment outcomes. Likewise, prior research has shown that NA is associated with morbidity and mortality (Piazza et al., 2013), including research in which “negative affect” was operationalized as depression (low PA according to the RCMA) (Wilson et al., 2003). Clinicians who misinterpret the term “negative affect” may inappropriately conflate depression (low PA) when developing interventions to improve health outcomes, while ignoring relevant high NA states (e.g., anger).

## Limitations

This study has many strengths, including the systematic literature search, the random selection of papers from a diverse array of high-impact journals representing different areas of psychology, and the use of two independent coders. In addition, we were conservative in our coding approach, such that we only coded papers as subscribing to the RCMA (and thus demonstrating orthogonality of PA and NA) if they (1) explicitly stated their use of RCMA, (2) cited Watson in reference to the conceptualization of affect, or (3) used a PANAS or PANAS-based measure. Many additional papers (63% of all papers) made statements that referred to PA and NA as independent/distinct constructs, and, thus, may have also assumed orthogonality. Therefore, the current findings may underestimate the pervasiveness of the “received view.”

It is also important to acknowledge several limitations and directions for future research. First, we opted to select papers from the highest impact journals in each sub-discipline area because these journals are likely most influential across the field; however, the extent to which these findings generalize to lower-impact journals remains unclear. The present approach was, however, deliberate, as we wanted to estimate the pervasiveness of the “received view” in the highest impact journals with presumably the highest standards for publication. Second, additional work is needed to determine the extent to which the misinterpretation of PA and NA as conceptualized in the RCMA extends to fields outside of psychology (e.g., medicine, public health, biology). Third, although we used a data-extraction template and two independent coders, the results remain susceptible to bias. It is also important to know that the findings of the present review have no bearing on the validity of the RCMA or the PANAS. Likewise, while there is an open empirical debate about the independence of positive and negative valence (i.e., pleasure and displeasure) in terms of psychological experience and underlying neurobiology (e.g., Feldman Barrett and Russell, 1998; Posner et al., 2005), the present review has no bearing on that issue.

Finally, articles were categorized as subscribing to the RCMA, and therefore adopting the principle of PA-NA orthogonality, if they explicitly referred to the circumplex model, cited a RCMA foundational paper by Watson and colleagues, or used some version of the PANAS. It is likely that for at least some of the papers that were categorized as subscribing to the RCMA, the authors did not intend to adopt the RCMA principle of PA-NA orthogonality. For example, it is possible that some authors used the PANAS simply because it is a widely used measure of affect and did not necessarily adopt the orthogonality principle. Thus, one might argue that it is unfair to claim theoretical inconsistency (i.e., “the received view”) if the principle of orthogonality was not explicitly stated in an article. On the contrary, we believe that use of the PANAS constitutes an inherent acceptance of the PA-NA orthogonality principle given that this principle wholly undergirds the creation and nature of the PANAS measure and its validity. Likewise, to cite a foundational RCMA paper as one’s theoretical or conceptual foundation necessarily assumes the PA-NA orthogonality principle as that is central to the RCMA.

Importantly, it is not our intention to criticize the specific authors whose research happened to have been randomly selected. We anticipate that, based on the systematic methods used, these findings should extend to additional articles, published by additional authors, in the psychological literature. Indeed, the pervasiveness of the “received view” makes clear that it is the result of a confluence of factors that has led to a systemic and widespread misinterpretation of the RCMA, rather than carelessness of a small number of researchers.

Moreover, the present study should not be interpreted as a critique of the RCMA or its authors. Indeed, the lead author of the RCMA has further developed the underlying theory to further focus on the interrelations among affect, motivation, personality, and psychopathology (e.g., Watson et al., 1992; Watson and Naragon-Gainey, 2014). Nonetheless, the RCMA has had a sustained impact on the field as has, unfortunately, its misinterpretation.

## Conclusion and recommendations

The confusion surrounding the interpretation of affect in accordance with the RCMA is well-documented and rampant throughout the psychological literature. Due to this confusion, the terms *positive affect* and *negative affect* should no longer be used. Consistent with Watson and Tellegen (1999) recommendation, the two dimensions of the RCMA and the corresponding subscales of the PANAS should be referred to as *positive activation* and *negative activation* (not *positive affect* and *negative affect*), to emphasize that they represent a union of positive valence and high activation and negative valence and high activation, respectively. Further, we recommend that the two poles of the valence dimension of the UCMA be referred to as positive valence and negative valence (or pleasure and displeasure; Russell, 1980), as opposed to PA and NA (to avoid confusion with RCMA). In summary, academic interest in, and empirical evaluation of the role of affect continues to grow. To advance our understanding of the underlying processes and role of affect in human behavior, it is critical that there is consistency in the use and application of major theories of affect.

## Author contributions

LL: Data curation, Investigation, Methodology, Writing – original draft. LC: Investigation, Methodology, Writing – review & editing. DW: Conceptualization, Methodology, Supervision, Writing – original draft, Writing – review & editing.
