# Supplementary material for: There is no happiness in positive affect: the pervasive misunderstanding of the rotated circumplex model
Source: Front Psychol. 2024 Mar 28;15:1301428. doi: 10.3389/fpsyg.2024.1301428 (PMC11007216; doi:10.3389/fpsyg.2024.1301428)
Supplement: Supplementary file 1 [file Data_Sheet_1.docx]

**SUPPLEMENTARY MATERIAL**

Appendix A: Reference List of Articles Randomly Selected for Inclusion in the Review

Appendix B: NVivo Extraction Instructions

Appendix C: Coding Domains/Guidelines

**Appendix A: Reference List of Articles Randomly Selected for Inclusion in the Review**

Aan Het Rot, M., Hogenelst, K., & Gesing, C. M. (2014). Communal and agentic behaviour in response to facial emotion expressions. *British Journal of Psychology*, *105*(2), 173-186.

Albarracin, D., & Hart, W. (2011). Positive mood+ action= negative mood+ inaction: effects of general action and inaction concepts on decisions and performance as a function of affect. *Emotion*, *11*(4), 951.

Allan, B. A., Steger, M. F., & Shin, J. Y. (2013). Thanks? Gratitude and well-being over the Thanksgiving holiday among college students. *The Journal of Positive Psychology*, *8*, 91-102.

Anker, J. J., Forbes, M. K., Almquist, Z. W., Menk, J. S., Thuras, P., Unruh, A. S., & Kushner, M. G. (2017). A network approach to modeling comorbid internalizing and alcohol use disorders. *Journal of Abnormal Psychology*, *126*(3), 325-339.

Ashton-James, C. E., Kushlev, K., & Dunn, E. W. (2013). Parents reap what they sow: Child-centrism and parental well-being. *Social Psychological and Personality Science*, *4*(6), 635-642.

Ashton-James, C. E., Maddux, W. W., Galinsky, A. D., & Chartrand, T. L. (2009). Who I am depends on how I feel: The role of affect in the expression of culture. *Psychological Science*, *20*(3), 340-346.

Ask, K., & Pina, A. (2011). On being angry and punitive: How anger alters perception of criminal intent. *Social Psychological and Personality Science*, *2*(5), 494-499.

Bandura, A., Caprara, G. V., Barbaranelli, C., Gerbino, M., & Pastorelli, C. (2003). Role of affective self‐regulatory efficacy in diverse spheres of psychosocial functioning. *Child Development*, *74*(3), 769-782.

Bar-Kalifa, E., Pshedetzky-Shochat, R., Rafaeli, E., & Gleason, M. E. (2018). Daily support equity in romantic couples: Response surface analyses of monadic and dyadic data. *Social Psychological and Personality Science*, *9*(7), 790-801.

Barnes, C. M., Miller, J. A., & Bostock, S. (2017). Helping employees sleep well: Effects of cognitive behavioral therapy for insomnia on work outcomes. *Journal of Applied Psychology*, *102*(1), 104-113.

Bartholow, B. D., Fabiani, M., Gratton, G., & Bettencourt, B. A. (2001). A psychophysiological examination of cognitive processing of and affective responses to social expectancy violations. *Psychological Science*, *12*(3), 197-204.

Bartolomeo, L. A., Culbreth, A. J., Ossenfort, K. L., & Strauss, G. P. (2020). Neurophysiological evidence for emotion regulation impairment in schizophrenia: The role of visual attention and cognitive effort. *Journal of Abnormal Psychology*, *129*(6), 670-676.

Bastiaansen, J. A., Ornée, D. A., Meurs, M., & Oldehinkel, A. J. (2022). An evaluation of the efficacy of two add-on ecological momentary intervention modules for depression in a pragmatic randomized controlled trial (ZELF-i). *Psychological Medicine*, *52*(13), 2731-2740.

Bauer, M. A., Wilkie, J. E., Kim, J. K., & Bodenhausen, G. V. (2012). Cuing consumerism: Situational materialism undermines personal and social well-being. *Psychological Science*, *23*(5), 517-523.

Bazhenova, O. V., Plonskaia, O., & Porges, S. W. (2001). Vagal reactivity and affective adjustment in infants during interaction challenges. *Child Development*, *72*(5), 1314-1326.

Becker, S. P., Tamm, L., Epstein, J. N., & Beebe, D. W. (2020). Impact of sleep restriction on affective functioning in adolescents with attention‐deficit/hyperactivity disorder. *Journal of Child Psychology and Psychiatry*, *61*(10), 1160-1168.

Böckler, A., Tusche, A., & Singer, T. (2016). The structure of human prosociality: Differentiating altruistically motivated, norm motivated, strategically motivated, and self-reported prosocial behavior. *Social Psychological and Personality Science*, *7*(6), 530-541.

Bonanno, G. A., Rennicke, C., & Dekel, S. (2005). Self-enhancement among high-exposure survivors of the September 11th terrorist attack: Resilience or social maladjustment? *Journal of Personality and Social Psychology*, *88*(6), 984-989.

Bos, E. H., de Jonge, P., & Cox, R. F. (2019). Affective variability in depression: Revisiting the inertia–instability paradox. *British Journal of Psychology*, *110*(4), 814-827.

Brewer, N. T., Parada Jr, H., Hall, M. G., Boynton, M. H., Noar, S. M., & Ribisl, K. M. (2019). Understanding why pictorial cigarette pack warnings increase quit attempts. *Annals of Behavioral Medicine*, *53*(3), 232-243.

Bridgett, D. J., Oddi, K. B., Laake, L. M., Murdock, K. W., & Bachmann, M. N. (2013). Integrating and differentiating aspects of self-regulation: effortful control, executive functioning, and links to negative affectivity. *Emotion*, *13*(1), 47-63.

Brown, L. H., Silvia, P. J., Myin-Germeys, I., & Kwapil, T. R. (2007). When the need to belong goes wrong: The expression of social anhedonia and social anxiety in daily life. *Psychological Science*, *18*(9), 778-782.

Busseri, M. A., Choma, B. L., & Sadava, S. W. (2012). Subjective temporal trajectories for subjective well-being. *The Journal of Positive Psychology*, *7*(1), 1-15.

Calderwood, C., & Ackerman, P. L. (2019). Modeling intraindividual variation in unsafe driving in a naturalistic commuting environment. *Journal of Occupational Health Psychology*, *24*(4), 423-437.

Chan, S. W., Lau, J. Y., & Reynolds, S. A. (2015). Is cognitive bias modification training truly beneficial for adolescents? *Journal of Child Psychology and Psychiatry*, *56*(11), 1239-1248.

Charles, S. T., Piazza, J. R., Mogle, J., Sliwinski, M. J., & Almeida, D. M. (2013). The wear and tear of daily stressors on mental health. *Psychological Science*, *24*(5), 733-741.

Cheeks, B. L., Chavous, T. M., & Sellers, R. M. (2020). A daily examination of African American adolescents’ racial discrimination, parental racial socialization, and psychological affect. *Child Development*, *91*(6), 2123-2140.

Chester, D. S., & Dzierzewski, J. M. (2020). Sour sleep, sweet revenge? Aggressive pleasure as a potential mechanism underlying poor sleep quality’s link to aggression. *Emotion*, *20*(5), 842-853.

Ciarrochi, J., Parker, P., Kashdan, T. B., Heaven, P. C., & Barkus, E. (2015). Hope and emotional well-being: A six-year study to distinguish antecedents, correlates, and consequences. *The Journal of Positive Psychology*, *10*(6), 520-532.

Cikara, M., & Fiske, S. T. (2012). Stereotypes and schadenfreude: Affective and physiological markers of pleasure at outgroup misfortunes. *Social Psychological and Personality Science*, *3*(1), 63-71.

Conner, T. S., DeYoung, C. G., & Silvia, P. J. (2018). Everyday creative activity as a path to flourishing. *The Journal of Positive Psychology*, *13*(2), 181-189.

Cousin, G., & Crane, C. (2016). Changes in disengagement coping mediate changes in affect following mindfulness‐based cognitive therapy in a non‐clinical sample. *British Journal of Psychology*, *107*(3), 434-447.

Creswell, K. G., Chung, T., Clark, D. B., & Martin, C. S. (2014). Solitary alcohol use in teens is associated with drinking in response to negative affect and predicts alcohol problems in young adulthood. *Clinical Psychological Science*, *2*(5), 602-610.

Cribbet, M. R., Williams, P. G., Gunn, H. E., & Rau, H. K. (2011). Effects of tonic and phasic respiratory sinus arrhythmia on affective stress responses. *Emotion*, *11*(1), 188-193.

Crosswell, A. D., Coccia, M., & Epel, E. S. (2020). Mind wandering and stress: When you don’t like the present moment. *Emotion*, *20*(3), 403-412.

Curhan, K. B., Levine, C. S., Markus, H. R., Kitayama, S., Park, J., Karasawa, M., Kawakami, N., Love, G. D., Coe, C. L., & Miyamoto, Y. (2014). Subjective and objective hierarchies and their relations to psychological well-being: A US/Japan comparison. *Social Psychological and Personality Science*, *5*(8), 855-864.

Cvetkovska, S., Verkuyten, M., Adelman, L., & Yogeeswaran, K. (2021). Being tolerated: Implications for well‐being among ethnic minorities. *British Journal of Psychology*, *112*(3), 781-803.

Czarna, A. Z., Wróbel, M., Dufner, M., & Zeigler-Hill, V. (2015). Narcissism and emotional contagion: Do narcissists “catch” the emotions of others? *Social Psychological and Personality Science*, *6*(3), 318-324.

Dagys, N., McGlinchey, E. L., Talbot, L. S., Kaplan, K. A., Dahl, R. E., & Harvey, A. G. (2012). Double trouble? The effects of sleep deprivation and chronotype on adolescent affect. *Journal of Child Psychology and Psychiatry*, *53*(6), 660-667.

Davey, C. G., Whittle, S., Harrison, B. J., Simmons, J. G., Byrne, M. L., Schwartz, O. S., & Allen, N. B. (2015). Functional brain-imaging correlates of negative affectivity and the onset of first-episode depression. *Psychological Medicine*, *45*(5), 1001-1009.

Davidov, M., & Grusec, J. E. (2006). Untangling the links of parental responsiveness to distress and warmth to child outcomes. *Child Development*, *77*(1), 44-58.

Davies, P. T., Coe, J. L., Hentges, R. F., Sturge-Apple, M. L., & Ripple, M. T. (2020). Temperamental Emotionality Attributes as Antecedents of Children's Social Information Processing. *Child Development*, *91*(2), 508-526.

de Groot, J. H., Smeets, M. A., Rowson, M. J., Bulsing, P. J., Blonk, C. G., Wilkinson, J. E., & Semin, G. R. (2015). A sniff of happiness. *Psychological Science*, *26*(6), 684-700.

de Hoog, N., & Verboon, P. (2020). Is the news making us unhappy? The influence of daily news exposure on emotional states. *British Journal of Psychology*, *111*(2), 157-173.

de Manzano, O., Theorell, T., Harmat, L., & Ullén, F. (2010). The psychophysiology of flow during piano playing. *Emotion*, *10*(3), 301-311.

De Saedeleer, L., & Pourtois, G. (2016). Evaluative priming reveals dissociable effects of cognitive versus physiological anxiety on action monitoring. *Emotion*, *16*(4), 498-514.

Deater-Deckard, K. (2000). Parenting and child behavioral adjustment in early childhood: a quantitative genetic approach to studying family processes. *Child Development*, *71*(2), 468-484.

Dejonckheere, E., Mestdagh, M., Houben, M., Erbas, Y., Pe, M., Koval, P., Brose, A., Bastian, B., & Kuppens, P. (2018). The bipolarity of affect and depressive symptoms. *Journal of Personality and Social Psychology*, *114*(2), 323-341.

Dekker, N., Smeerdijk, A. M., Wiers, R. W., Duits, J. H., van Gelder, G., Houben, K., Schippers, G., Linszen, D. H., & de Haan, L. (2010). Implicit and explicit affective associations towards cannabis use in patients with recent-onset schizophrenia and healthy controls. *Psychological Medicine*, *40*(8), 1325-1336.

Diefendorff, J. M., Gabriel, A. S., Nolan, M. T., & Yang, J. (2019). Emotion regulation in the context of customer mistreatment and felt affect: An event-based profile approach. *Journal of Applied Psychology*, *104*(7), 965-983.

Dimotakis, N., Davison, R. B., & Hollenbeck, J. R. (2012). Team structure and regulatory focus: the impact of regulatory fit on team dynamic. *Journal of Applied Psychology*, *97*(2), 421-434.

Donnelly, G. E., Zatz, L. Y., Svirsky, D., & John, L. K. (2018). The Effect of Graphic Warnings on Sugary-Drink Purchasing. *Psychological Science*, *29*(8), 1321-1333.

Dunton, G. F., Atienza, A. A., Castro, C. M., & King, A. C. (2009). Using ecological momentary assessment to examine antecedents and correlates of physical activity bouts in adults age 50+ years: a pilot study. *Annals of Behavioral Medicine*, *38*(3), 249-255.

Echteld, M. A., van Elderen, T., & van der Kamp, L. J. (2003). Modeling predictors of quality of life after coronary angioplasty. *Annals of Behavioral Medicine*, *26*(1), 49-60.

Eckstrand, K. L., Hanford, L. C., Bertocci, M. A., Chase, H. W., Greenberg, T., Lockovich, J., Stiffler, R., Aslam, H. A., Graur, S., Bebko, G., Forbes, E. E., & Phillips, M. L. (2019). Trauma-associated anterior cingulate connectivity during reward learning predicts affective and anxiety states in young adults. *Psychological Medicine*, *49*(11), 1831-1840.

Eisenkraft, N., & Elfenbein, H. A. (2010). The way you make me feel: evidence for individual differences in affective presence. *Psychological Science*, *21*(4), 505-510.

Elavsky, S., & McAuley, E. (2007). Physical activity and mental health outcomes during menopause: a randomized controlled trial. *Annals of Behavioral Medicine*, *33*(2), 132-142.

Elavsky, S., McAuley, E., Motl, R. W., Konopack, J. F., Marquez, D. X., Hu, L., Jerome, G. J., & Diener, E. (2005). Physical activity enhances long-term quality of life in older adults: efficacy, esteem, and affective influences. *Annals of Behavioral Medicine*, *30*(2), 138-145.

Erez, A., Misangyi, V. F., Johnson, D. E., LePine, M. A., & Halverson, K. C. (2008). Stirring the hearts of followers: Charismatic leadership as the transferal of affect. *Journal of Applied Psychology*, *93*(3), 602-616.

Ezpeleta, L., Granero, R., de la Osa, N., Penelo, E., & Domènech, J. M. (2012). Dimensions of oppositional defiant disorder in 3-year-old preschoolers. *Journal of Child Psychology and Psychiatry*, *53*(11), 1128-1138.

Falkenstern, M., Schiffrin, H. H., Nelson, S. K., Ford, L., & Keyser, C. (2009). Mood over matter: can happiness be your undoing? *The Journal of Positive Psychology*, *4*(5), 365-371.

Foo, M. D., Uy, M. A., & Baron, R. A. (2009). How do feelings influence effort? An empirical study of entrepreneurs' affect and venture effort. *Journal of Applied Psychology*, *94*(4), 1086-1094.

Forgeard, M., Beard, C., Shayani, D., Silverman, A. L., Tsukayama, E., & Björgvinsson, T. (2021). Predictors of affect following discharge from partial hospitalization: a two-week ecological momentary assessment study. *Psychological Medicine*, *51*(7), 1157-1165.

Froh, J. J., Kashdan, T. B., Ozimkowski, K. M., & Miller, N. (2009). Who benefits the most from a gratitude intervention in children and adolescents? Examining positive affect as a moderator. *The Journal of Positive Psychology*, *4*(5), 408-422.

Gerhart, J. I., Burns, J. W., Post, K. M., Smith, D. A., Porter, L. S., Burgess, H. J., Schuster, E., Buvanendran, A., Fras, A. M., & Keefe, F. J. (2017). Relationships Between Sleep Quality and Pain-Related Factors for People with Chronic Low Back Pain: Tests of Reciprocal and Time of Day Effects. *Annals of Behavioral Medicine*, *51*(3), 365-375.

Geva, R., Schreiber, J., Segal-Caspi, L., & Markus-Shiffman, M. (2014). Neonatal brainstem dysfunction after preterm birth predicts behavioral inhibition. *Journal of Child Psychology and Psychiatry*, *55*(7), 802-810.

Gibbons, F. X., Lane, D. J., Gerrard, M., Reis-Bergan, M., Lautrap, C. L., Pexa, N. A., & Blanton, H. (2002). Comparison-level preferences after performance: is downward comparison theory still useful? *Journal of Personality and Social Psychology*, *83*(4), 865-880.

Hanson, E. K., Maas, C. J., Meijman, T. F., & Godaert, G. L. (2000). Cortisol secretion throughout the day, perceptions of the work environment, and negative affect. *Annals of Behaivoral Medicine*, *22*(4), 316-324.

Hewig, J., Hagemann, D., Seifert, J., Naumann, E., & Bartussek, D. (2004). On the selective relation of frontal cortical asymmetry and anger-out versus anger-control. *Journal of Personality and Social Psychology*, *87*(6), 926-939.

Hill, P. L., & Allemand, M. (2011). Gratitude, forgivingness, and well-being in adulthood: Tests of moderation and incremental prediction. *The Journal of Positive Psychology*, *6*(5), 397-407.

Hutchison, K. E., LaChance, H., Niaura, R., Bryan, A., & Smolen, A. (2002). The DRD4 VNTR polymorphism influences reactivity to smoking cues. *Journal of Abnormal Psychology*, *111*(1), 134-143.

Ilies, R., Dimotakis, N., & Watson, D. (2010). Mood, blood pressure, and heart rate at work: an experience-sampling study. *Journal of Occupational Health Psychology*, *15*(2), 120-130.

Jamieson, J. P., Nock, M. K., & Mendes, W. B. (2013). Changing the Conceptualization of Stress in Social Anxiety Disorder: Affective and Physiological Consequences. *Clinical Psychological Science*, *1*(4), 363-374.

Jaya, E. S., Ascone, L., & Lincoln, T. M. (2018). A longitudinal mediation analysis of the effect of negative-self-schemas on positive symptoms via negative affect. *Psychological Medicine*, *48*(8), 1299-1307.

Jenkins, B. N., Hunter, J. F., Richardson, M. J., Conner, T. S., & Pressman, S. D. (2020). Affect variability and predictability: Using recurrence quantification analysis to better understand how the dynamics of affect relate to health. *Emotion*, *20*(3), 391-402.

Joh, A. S., & Adolph, K. E. (2006). Learning from falling. *Child Development*, *77*(1), 89-102.

Jones, F., O'Connor, D. B., Conner, M., McMillan, B., & Ferguson, E. (2007). Impact of daily mood, work hours, and iso-strain variables on self-reported health behaviors. *Journal of Applied Psychology*, *92*(6), 1731-1740.

Jungmann, S. M., Brand, S., Kolb, J., & Witthöft, M. (2020). Do Dr. Google and Health Apps Have (Comparable) Side Effects? An Experimental Study. *Clinical Psychological Science*, *8*(2), 306-317.

Kammerer, M. K., Mehl, S., Ludwig, L., & Lincoln, T. M. (2021). Sleep and circadian rhythm disruption predict persecutory symptom severity in day-to-day life: A combined actigraphy and experience sampling study. *Journal of Abnormal Psychology*, *130*(1), 78-88.

Kärtner, J., Crafa, D., Chaudhary, N., & Keller, H. (2016). Reactions to Receiving a Gift-Maternal Scaffolding and Cultural Learning in Berlin and Delhi. *Child Development*, *87*(3), 712-722.

Kehr, H. M. (2003). Goal conflicts, attainment of new goals, and well-being among managers. *Journal of Occupational and Health Psychology*, *8*(3), 195-208.

Kim, S., Park, Y., & Headrick, L. (2018). Daily micro-breaks and job performance: General work engagement as a cross-level moderator. *Journal of Applied Psychology*, *103*(7), 772-786.

Koenig, A. L., Cicchetti, D., & Rogosch, F. A. (2000). Child compliance/noncompliance and maternal contributors to internalization in maltreating and nonmaltreating dyads. *Child Development*, *71*(4), 1018-1032.

Koopman, J., Conway, J. M., Dimotakis, N., Tepper, B. J., Lee, Y. E., Rogelberg, S. G., & Lount, R. B. (2021). Does CWB repair negative affective states, or generate them? Examining the moderating role of trait empathy. *Journal of Applied Psychology*, *106*(10), 1493-1516.

Kovacs, M., Bylsma, L. M., Yaroslavsky, I., Rottenberg, J., George, C. J., Kiss, E., Halas, K., Benák, I., Baji, I., & Vetró, Á. (2016). Positive affectivity is dampened in youths with histories of major depression and their never-depressed adolescent siblings. *Clinical Psychological Science*, *4*(4), 661-674.

Krick, A., & Felfe, J. (2020). Who benefits from mindfulness? The moderating role of personality and social norms for the effectiveness on psychological and physiological outcomes among police officers. *Journal of Occupational and Health Psychology*, *25*(2), 99-112.

Landa, R. J., Holman, K. C., O'Neill, A. H., & Stuart, E. A. (2011). Intervention targeting development of socially synchronous engagement in toddlers with autism spectrum disorder: a randomized controlled trial. *Journal of Child Psychology and Psychiatry*, *52*(1), 13-21.

Leach, S., & Weick, M. (2020). When smiles (and frowns) speak words: Does power impact the correspondence between self-reported affect and facial expressions? *British Journal of Psychology*, *111*(4), 683-701.

Lee, R. R., & Chatzisarantis, N. L. D. (2017). Same but different: Comparative modes of information processing are implicated in the construction of perceptions of autonomy support. *British Journal of Psychology*, *108*(4), 687-700.

Lüscher, J., Hohl, D. H., Knoll, N., & Scholz, U. (2019). Invisible Social Support and Invisible Social Control in Dual-smoker Couple's Everyday Life: A Dyadic Perspective. *Annals of Behavioral Medicine*, *53*(6), 527-540.

Maher, J. P., Ra, C. K., Leventhal, A. M., Hedeker, D., Huh, J., Chou, C. P., & Dunton, G. F. (2018). Mean level of positive affect moderates associations between volatility in positive affect, mental health, and alcohol consumption among mothers. *Journal of Abnormal Psychology*, *127*(7), 639-649.

Maltby, J., Day, L., Hatcher, R. M., Tazzyman, S., Flowe, H. D., Palmer, E. J., Frosch, C. A., O'Reilly, M., Jones, C., Buckley, C., Knieps, M., & Cutts, K. (2016). Implicit theories of online trolling: Evidence that attention-seeking conceptions are associated with increased psychological resilience. *British Journal of Psychology*, *107*(3), 448-466.

Mora, P. A., DiBonaventura, M. D., Idler, E., Leventhal, E. A., & Leventhal, H. (2008). Psychological factors influencing self-assessments of health: toward an understanding of the mechanisms underlying how people rate their own health. *Annals of Behavioral Medicine*, *36*(3), 292-303.

Myin-Germeys, I., Krabbendam, L., Delespaul, P. A., & Van Os, J. (2003). Do life events have their effect on psychosis by influencing the emotional reactivity to daily life stress? *Psychological Medicine*, *33*(2), 327-333.

Naragon-Gainey, K., & DeMarree, K. G. (2017). Decentering attenuates the associations of negative affect and positive affect with psychopathology. *Clinical Psychological Science*, *5*(6), 1027-1047.

Nelson, B. W., Byrne, M. L., Sheeber, L., & Allen, N. B. (2017). Does Context Matter? A Multi-Method Assessment of Affect in Adolescent Depression Across Multiple Affective Interaction Contexts. *Clinical Psychological Science*, *5*(2), 239-258.

Newman, M. G., Jacobson, N. C., Zainal, N. H., Shin, K. E., Szkodny, L. E., & Sliwinski, M. J. (2019). The Effects of Worry in Daily Life: An Ecological Momentary Assessment Study Supporting the Tenets of the Contrast Avoidance Model. *Clinical Psychological Science*, *7*(4), 794-810.

Norris, C. J., & Wu, E. (2021). Accentuate the positive, eliminate the negative: Reducing ambivalence through instructed emotion regulation. *Emotion*, *21*(3), 499-512.

Oishi, S., Lun, J., & Sherman, G. D. (2007). Residential mobility, self-concept, and positive affect in social interactions. *Journal of Personality and Social Psychology*, *93*(1), 131-141.

Orth, U., Robins, R. W., & Widaman, K. F. (2012). Life-span development of self-esteem and its effects on important life outcomes. *Journal of Personality and Social Psychology*, *102*(6), 1271-1288.

Park, Y., & Kim, S. (2019). Customer mistreatment harms nightly sleep and next-morning recovery: Job control and recovery self-efficacy as cross-level moderators. *Journal of Occupational and Health Psychology*, *24*(2), 256-269.

Parke, M. R., Seo, M. G., & Sherf, E. N. (2015). Regulating and facilitating: the role of emotional intelligence in maintaining and using positive affect for creativity. *Journal of Applied Psychology*, *100*(3), 917-934.

Rademaker, A. R., van Zuiden, M., Vermetten, E., & Geuze, E. (2011). Type D personality and the development of PTSD symptoms: a prospective study. *Journal of Abnormal Psychology*, *120*(2), 299-307.

Reh, S., Wieck, C., & Scheibe, S. (2021). Experience, vulnerability, or overload? Emotional job demands as moderator in trajectories of emotional well-being and job satisfaction across the working lifespan. *Journal of Applied Psychology*, *106*(11), 1734-1749.

Reis, H. T., O'Keefe, S. D., & Lane, R. D. (2017). Fun Is More Fun When Others Are Involved. *Journal of Positive Psychology*, *12*(6), 547-557.

Rosenblum, K. L., McDonough, S., Muzik, M., Miller, A., & Sameroff, A. (2002). Maternal representations of the infant: associations with infant response to the still face. *Child Development*, *73*(4), 999-1015.

Ruedy, N. E., Moore, C., Gino, F., & Schweitzer, M. E. (2013). The cheater's high: the unexpected affective benefits of unethical behavior. *Journal of Personality and Social Psychology*, *105*(4), 531-548.

Sajjad, A., Freak-Poli, R. L., Hofman, A., Roza, S. J., Ikram, M. A., & Tiemeier, H. (2017). Subjective measures of health and all-cause mortality - the Rotterdam Study. *Psychological Medicine*, *47*(11), 1971-1980.

Samios, C., Pakenham, K. I., & O'Brien, J. (2015). A dyadic and longitudinal investigation of adjustment in couples coping with multiple sclerosis. *Annals of Behavioral Medicine*, *49*(1), 74-83.

Santangelo, P. S., Holtmann, J., Hosoya, G., Bohus, M., Kockler, T. D., Koudela-Hamila, S., Eid, M., & Ebner-Priemer, U. W. (2020). Within- and Between-Persons Effects of Self-Esteem and Affective State as Antecedents and Consequences of Dysfunctional Behaviors in the Everyday Lives of Patients With Borderline Personality Disorder. *Clinical Psychological Science*, *8*(3), 428-449.

Saucier, G., Georgiades, S., Tsaousis, I., & Goldberg, L. R. (2005). The factor structure of Greek personality adjectives. *Journal of Personality and Social Psychology*, *88*(5), 856-875.

Schat, A. C. H., Kelloway, E. K., & Desmarais, S. (2005). The Physical Health Questionnaire (PHQ): construct validation of a self-report scale of somatic symptoms. *Journal of Occupational and Health Psychology*, *10*(4), 363-381.

Schmitt, A., Den Hartog, D. N., & Belschak, F. D. (2015). Is outcome responsibility at work emotionally exhausting? Investigating employee proactivity as a moderator. *Journal of Occupational and Health Psychology*, *20*(4), 491-500.

Schroevers, M. J., & Brandsma, R. (2010). Is learning mindfulness associated with improved affect after mindfulness-based cognitive therapy? *British Journal of Psychology*, *101*(Pt 1), 95-107.

Schuetze, P., Eiden, R. D., & Danielewicz, S. (2009). The association between prenatal cocaine exposure and physiological regulation at 13 months of age. *Journal of Child Psychology and Psychiatry*, *50*(11), 1401-1409.

Scott, L. N., Wright, A. G. C., Beeney, J. E., Lazarus, S. A., Pilkonis, P. A., & Stepp, S. D. (2017). Borderline personality disorder symptoms and aggression: A within-person process model. *Journal of Abnormal Psychology*, *126*(4), 429-440.

Smith, D. W., & Brodzinsky, D. M. (2002). Coping with birthparent loss in adopted children. *Journal of Child Psychology and Psychiatry*, *43*(2), 213-223.

Smith, T. W., Cribbet, M. R., Nealey-Moore, J. B., Uchino, B. N., Williams, P. G., Mackenzie, J., & Thayer, J. F. (2011). Matters of the variable heart: respiratory sinus arrhythmia response to marital interaction and associations with marital quality. *Journal of Personality and Social Psychology*, *100*(1), 103-119.

Sonnentag, S., & Lischetzke, T. (2018). Illegitimate tasks reach into afterwork hours: A multilevel study. *Journal of Occupational and Health Psychology*, *23*(2), 248-261.

Stellern, S., Esposito, E., Mliner, S., Pears, K., & Gunnar, M. (2014). Increased freezing and decreased positive affect in postinstitutionalized children. *Journal of Child Psychology and Psychiatry*, *55*(1), 88-95.

Steptoe, A., O'Donnell, K., Marmot, M., & Wardle, J. (2008). Positive affect and psychosocial processes related to health. *British Journal of Psychology*, *99*(Pt 2), 211-227.

Tan, J. J. X., Kraus, M. W., Impett, E. A., & Keltner, D. (2019). Partner Commitment in Close Relationships Mitigates Social Class Differences in Subjective Well-Being. *Social Psychological and Personality Science*, *11*(1), 16-25.

Thoman, D. B., Smith, J. L., & Silvia, P. J. (2011). The Resource Replenishment Function of Interest. *Social Psychological and Personality Science*, *2*(6), 592-599.

Tottenham, N., Phuong, J., Flannery, J., Gabard-Durnam, L., & Goff, B. (2013). A negativity bias for ambiguous facial-expression valence during childhood: converging evidence from behavior and facial corrugator muscle responses. *Emotion*, *13*(1), 92-103.

Unsworth, K. L., & Mason, C. M. (2012). Help yourself: the mechanisms through which a self-leadership intervention influences strain. *Journal of Occupational and Health Psychology*, *17*(2), 235-245.

Vallerand, R. J., Blanchard, C., Mageau, G. A., Koestner, R., Ratelle, C., Leonard, M., Gagne, M., & Marsolais, J. (2003). Les passions de l'ame: on obsessive and harmonious passion. *Journal of Personality and Social Psychology*, *85*(4), 756-767.

Wang, Q., Hou, Y., Koh, J. B. K., Song, Q., & Yang, Y. (2018). Culturally Motivated Remembering: The Moderating Role of Culture for the Relation of Episodic Memory to Well-Being. *Clinical Psychological Science*, *6*(6), 860-871.

Wenzel, M., Kubiak, T., & Conner, T. S. (2016). Self-Control in Daily Life: How Affect May Boost or Sabotage Efforts at Self-Control. *Social Psychological and Personality Science*, *7*(3), 195-203.

Wichers, M., Schrijvers, D., Geschwind, N., Jacobs, N., Myin-Germeys, I., Thiery, E., Derom, C., Sabbe, B., Peeters, F., Delespaul, P., & van Os, J. (2009). Mechanisms of gene-environment interactions in depression: evidence that genes potentiate multiple sources of adversity. *Psychological Medicine*, *39*(7), 1077-1086.

Williams-Kerver, G. A., Wonderlich, S. A., Crosby, R. D., Cao, L., Smith, K. E., Engel, S. G., Crow, S. J., Peterson, C. B., Mitchell, J. E., & Le Grange, D. (2020). Differences in Affective Dynamics Among Eating Disorder Diagnostic Groups. *Clinical Psychological Science*, *8*(5), 857-871.

Zheng, Y., Plomin, R., & von Stumm, S. (2016). Heritability of Intraindividual Mean and Variability of Positive and Negative Affect. *Psychological Science*, *27*(12), 1611-1619.

Zhou, Z. E., Yan, Y., Che, X. X., & Meier, L. L. (2015). Effect of workplace incivility on end-of-work negative affect: examining individual and organizational moderators in a daily diary study. *Journal of Occupational and Health Psychology*, *20*(1), 117-130.

**Appendix B: NVivo Extraction Instructions**

1. Find the measure(s) of affect
   1. Examine the measures section to determine which measure(s) of affect were used
   2. Extract and code all text that describes the first measure of affect as ‘Measure Description 1’ in NVivo
   3. If there are additional measure(s) of affect, code as ‘Measure Description 2’, ‘Measure Description 3’, etc. in Nvivo
      1. You may need to make additional ‘nodes’ in NVivo to accommodate this
   4. Code all positive affect terms as ‘PA Affective States Terms’ in NVivo
   5. Code all negative affect terms as ‘NA Affective States Terms’ in NVivo
   6. If a paper has multiple studies and the same measure was used in more than one study, extract the description from only the first study in which the measure was used (unless there is new/additional information in the measures section of other studies, in which case that text should also be extracted)
2. Search for a definition of affect using the search terms: “defin”, “affect is”, “PA is”, “NA is”
   1. If a clear conceptual definition is provided (typically in the introduction section), extract the statement and code as ‘Definition of Affect’ in NVivo
      1. Do not extract/code *operational* definitions (e.g., PA was defined as the sum of responses to…)
      2. If it is unclear whether the statement is a *conceptual* definition of affect, code instead as an ‘Other Statement on Affect’ (described in Step 5, below)
3. Search for references to “Watson” and/or “Russell”
   1. Code all statements that include citations for David Watson as ‘Watson Citation’ in NVivo and code the reference as ‘References for Watson Citation’
   2. Code all statements that include citations for James A. Russell as ‘Russell Citation’ in NVivo and code the reference as ‘References for Russell Citation’
4. Search for all instances of the word “circumplex”
   1. Code all statements that include the word “circumplex” as ‘Circumplex’ in NVivo
      1. Only extract/code statements that are applicable to affect (i.e., do not extract the statement if it is describing a different circumplex model, such as the circumplex model of marital and family systems)
5. Search for all instances of the terms: “affect”, “PA”, “NA”, “feeling”, “valence”
   1. Code all statements that refer to affect as ‘Other Statement on Affect’ in NVivo
      1. Do not extract statements that include the word “affective’” in way that does not directly relate to affect (e.g., affective disorders, affective reactivity)
      2. Be sure to include statements that occur in tables, figures, notes, and abstract
      3. Do not extract/code subtitles/section titles

**Notes*

- A ‘statement’ is typically a single sentence. Only extract more than one sentence if the additional sentence(s) are needed to provide context.
- All categories are independent (i.e., a single statement should not be coded in two different categories)

**Appendix C: Coding Domains/Guidelines**

**General Study Information**

1. Study ID:

2. Title:

3. Lead author email address:

4. Year:

5. Journal (select):

1. Journal of Positive Psychology
2. Journal of Child Psychology & Psychiatry
3. Journal of Occupational Health Psychology
4. British Journal of Psychology
5. Annals of Behavioral Medicine
6. Child Development
7. Clinical Psychological Science
8. Emotion
9. Journal of Abnormal Psychology
10. Journal of Applied Psychology
11. Journal of Personality and Social Psychology
12. Psychological Medicine
13. Psychological Science
14. Social Psychological and Personality Science

6. Study Design Type (select):

1. Observation, cross-sectional
2. Observational, longitudinal
3. Experimental
4. Qualitative

**Evidence of Subscribing to the RCMA**

1. Was the rotated circumplex model identified as the model of affect?

1. Yes
2. No

2. Was Watson cited in reference to the conceptualization of affect? (*Note: any measure that is referred to as “affect”, “positive affect”, or “negative affect” at any point in the methods or results is considered a measure of affect; measures that describe related but distinct constructs such as negative affectivity or affective disorders should not be coded*)

1. Yes
2. No

3. Was one of the following used to measure affect? (*Note: measures that reference the PANAS in the measures section [e.g., are described as ‘based on’, ‘including items from’, or ‘a modified version of’ the PANAS] and/or cite at least one of the PANAS versions should be coded as a modified version of the PANAS*)

1. PANAS
2. PANAS-X
3. PANAS-SF
4. I-PANAS-SF
5. Modified version of the PANAS

**Evidence of the View that PA = Positive Valence and NA = Negative Valence**

1. Of the negative valence states included in the measure of NA, were only high activation states included?

1. Yes
2. No, low activation states were included
3. No, neutral activation states (but no low activation states) were included
4. No example affect terms were provided, thus unable to assess

2. Of the positive valence states included in the measure of PA, were only high activation states included?

1. Yes
2. No, low activation states were included
3. No, neutral activation states (but no low activation states) were included
4. No example affect terms were provided, thus unable to assess

3. Were there any statement(s) in which the authors generalized PA or positive valence to all positively valence states, including at least one positive valence low arousal state?

1. Yes
2. No

4. Were there any statement(s) in which the authors generalized NA or negative valence to all negatively valenced states, including at least one negative valence low arousal state?

1. Yes
2. No

**Other**

1. Was a conceptual definition of affect provided?

1. Yes
2. No

2. Was the unrotated circumplex model identified as the model of affect?

1. Yes
2. No

3. Was Russell cited in reference to the conceptualization of affect?

1. Yes
2. No
